# Complications and TKA conversion rates in unicondylar vs. bicondylar tibial plateau fractures: a retrospective cohort analysis

**DOI:** 10.1007/s00402-025-06107-0

**Published:** 2025-10-29

**Authors:** Cyrus Eghtedari, Alexander Berk, Logan Good, Samuel Florentino, Robert Burkhart, Harkirat Jawanda, George Ochenjele, Robert Wetzel, Joshua Napora

**Affiliations:** 1 University Hospitals of Cleveland, Cleveland, USA; 2 Case Western Reserve University, Cleveland, USA

**Keywords:** Tibial plateau, Unicondylar, Bicondylar, Total knee arthroplasty, TKA, Complications

## Abstract

**Background:**

Tibial plateau fractures are associated with a high incidence of posttraumatic osteoarthritis. This study aims to compare the rates of medical and surgical complications, as well as the rate of conversion to total knee arthroplasty (TKA), among patients undergoing operative fixation of tibial plateau fractures.

**Methods:**

The TriNetX database was queried to identify patients aged 18 and older who underwent operative fixation of unicondylar (CPT 27535) and bicondylar (CPT 27536) tibial plateau fractures between January 2005 and January 2020. Patients with bilateral fractures, and those with a prior history of TKA were excluded. Medical and surgical complications were analyzed during the immediate (7d, 14d, and 30d), intermediate (90d, 180d, 1y, and 2y), and long-term (5y and 10y) postoperative periods. A multivariate model, adjusted for age, sex, and comorbidities, was used to further evaluate the risk of conversion to TKA.

**Results:**

A total of 15,634 patients met all inclusion criteria and were included in this study (8,680 unicondylar and 6,954 bicondylar). Bivariate analysis revealed a higher risk of medical complications including DVT, PE, pneumonia, and death in the bicondylar group. The risk of surgical complications including superficial skin infection, deep infection, rehospitalization, arthrofibrosis, malunion, and nonunion were also higher in the bicondylar group. No differences were found in the rates of MI, stroke, cardiac arrest, pneumonia, ARDS, ED visits, or UTIs between groups at any time point. After multivariate analysis, the risk of conversion TKA was greater in the bicondylar fracture group at 5y (HR [95% CI] 1.43 [1.14, 1.79], *p* = 0.002) and 10y (HR [95% CI] 1.40 [1.14, 1.72], *p* = 0.001) follow-up.

**Conclusions:**

Patients undergoing operative fixation of bicondylar tibial plateau fractures experience higher rates of medical and surgical complications, as well as an increased incidence of conversion to TKA, compared to those with unicondylar fractures.

**Level of evidece:**

Prognostic Level III.

**Supplementary Information:**

The online version contains supplementary material available at 10.1007/s00402-025-06107-0.

## Introduction

Tibial plateau fractures (TPFs) are multifaceted intra-articular injuries that disrupt the proximal tibia, often leading to significant morbidity and a compromised quality of life due to pain, stiffness, and post-traumatic arthritis (PTOA) [1]. Open reduction and internal fixation (ORIF) prevails as the gold standard treatment with the goal of restoring joint stability and alignment [2, 3]. However, patients remain at risk for long-term complications including PTOA requiring conversion total knee arthroplasty (TKA) [3, 4]. The Schatzker classification system categorizes TPFs based on their anatomical involvement, with unicondylar fractures affecting a single condyle and bicondylar fractures involving both the medial and lateral condyles. Unicondylar tibial plateau fractures, which include Schatzker types I-III, are often linked to lower-energy mechanisms, whereas bicondylar tibial plateau fractures, types IV-VI, are associated with higher-energy mechanisms [5]. In recognizing that these subtypes differ in biomechanical stability and soft tissue involvement, clinicians are enabled to balance the goal of restoring joint congruity with the risk of PTOA and eventual need for TKA [6–8].

While both unicondylar and bicondylar fractures can lead to joint degeneration, current literature remains inconsistent in comparing ORIF complications and TKA conversion rates between unicondylar and bicondylar TPFs. Ruffolo et al.identified a notable incidence in major complications amongst bicondylar fractures, highlighting deep infections and nonunion as significant issues [9]. In contrast, Olszewski et al.and Unno et al.observed lower infection rates in similar fractures, suggesting variability in outcomes [10, 11]. Moreover, Wasserstein et al.and Kraml et al.both found that a meaningful proportion of tibial plateau fracture cases progressed to TKA over a decade, with differing emphases on the long-term conversion risk [12, 13]. Although, studies have suggested that bicondylar fractures carry a heightened risk due to increased injury severity and comorbidities, direct comparisons between unicondylar and bicondylar fractures are limited, necessitating further exploration of their respective clinical courses.

Given the prevalence and morbidity of TPFs, there remains a need to better understand the prognostic implications associated with particular fracture types. To our knowledge, no prior study has compared the outcomes and complications of bicondylar TPFs to unicondylar TPFs using a large national multicenter database. An expanded understanding of the outcomes of these injuries could aid in surgeon decision making and enable more comprehensive discussions with patients regarding prognosis and postoperative expectations. Thus, the purpose of this study is to compare the rates of medical and surgical complications, as well as the incidence of conversion TKA, between patients with operatively treated unicondylar and bicondylar tibial plateau fractures.

## Methods

### Study design

The TriNetX United States Collaborative Network (Cambridge, Massachusetts, United States) was used to perform a retrospective cohort study which includes aggregated and deidentified data from more than 118 million patients and 68 Health Care Organizations (HCOs) [14]. The platform incorporates data from both insured and uninsured patients from a variety of organizations including hospitals as well as primary and specialty care centers. Available data includes demographics, diagnoses, procedures, medications, laboratory values, and genomic information. Through access to real-time clinical data, the database facilitates the analysis of large, complex data sets thereby enhancing the robustness of study findings. Institutional Review Board (IRB) approval was not required for this study as TriNetX abides by Health Insurance Portability and Accountability Act (HIPAA) and General Data Protection Regulation (GDPR) guidelines for deidentification.

### Study population

The TriNetX US Collaborative Network database was queried using International Classification of Disease, 10th Revision (ICD-10) and Current Procedural Terminology (CPT) codes to identify patients aged 18 and older who underwent operative fixation of a unicondylar (CPT 27535) or bicondylar (CPT 27536) TPF between 2005 and 2020 (Supplementary Digital Content 1). Exclusion criteria included patients with bilateral fractures and those with a history of prior TKA.

### Patient demographics

A total of 15,634 patients met all inclusion criteria and were included in the study. Among these, 8,680 (56%) sustained an operatively managed unicondylar tibial plateau fracture, while 6,954 (44%) sustained a bicondylar fracture. Prior to risk-adjusted analysis, the bicondylar fracture group was significantly (p *≤* 0.05) older and had a higher prevalence of primary hypertension. In contrast, the unicondylar group had higher rates of nicotine dependence and was more likely to be female, Black, or Hispanic/Latino (Table 1).

### Data collection and outcomes

Tibial was identified as the index event. Patient cohorts were evaluated for demographics, including age, race, ethnicity, and sex, as well as comorbidities. Medical outcomes and complications were assessed at multiple time points up to one year, while surgical outcomes and complications were evaluated through two years. The risk of conversion to TKA was assessed at 1-, 2-, 5, and 10-years. Medical complications evaluated included deep vein thrombosis (DVT), pulmonary embolism (PE), myocardial infarction (MI), stroke, cardiac arrest, pneumonia, acute kidney injury (AKI), urinary tract infection (UTI), acute respiratory distress syndrome (ARDS), sepsis, and death. Surgical complications assessed included superficial skin infection (SSI), deep infection, Emergency Department (ED) visits, rehospitalization, and arthrofibrosis requiring manipulation under anesthesia or lysis of adhesions. Further, the risk of malunion and nonunion were evaluated starting at 180d postoperatively.

### Statistical analysis

The TriNetX platform was utilized for all statistical analyses using built-in statistical tools. Descriptive statistics were calculated for all continuous and categorical variables. Continuous variables were reported as mean ± standard deviation (SD), whereas categorical variables were reported as frequencies with percentages. A chi-squared (χ^2^) test was used to analyze categorical variables, and an independent samples t-test was used to analyze the difference in means for continuous variables. A multivariate model adjusted for age, sex, and comorbidities was used to further evaluate the risk of conversion to TKA. Risk values were reported as relative risk (RR) with a 95% confidence interval (CI). A p-value *≤* 0.05 was considered statistically significant.

## Results

### Medical outcomes and complications

Amongst both cohorts the most common medical complications were DVT, UTI, AKI and pneumonia (Table 2). The risk of DVT and PE were greater in the bicondylar group at all time points, with one-year relative risks of 1.27 [1.09,1.48; *p* = 0.002] and 1.33 [1.06,1.668; *p* = 0.013], respectively. The risk of pneumonia was also higher in this group at 30 days (RR 1.27 [1.02,1.59]; *p* = 0.003) and 90 days (RR 1.22 [1.01,1.49]; *p* = 0.043). Additionally, the bicondylar cohort had an increased risk of AKI and sepsis at 90 days, 180 days, and one year, with one-year relative risks of 1.25 [1.06,1.48; *p* = 0.004] and 1.36 [1.11,1.68; *p* = 0.004], respectively. Lastly, mortality risk was significantly higher in the bicondylar group at 180 days (RR 1.44 [1.08,1.93]; *p* = 0.013) and one year (RR 1.46 [1.15,1.86]; *p* = 0.002). No significant difference was observed in the rates of MI, stroke, cardiac arrest, UTI, or ARDS at any time point.

**Table 1:** Unicondylar vs. Bicondylar Tibial Plateau Fracture Patient Demographics*

	Unicondylar (*n* = 8,680)	Bicondylar (*n* = 6,954)	*p-*value
Demographics			
Age, y	45.6 ± 17.5	49.2 ± 15.9	**< 0.001**
Female	3,342 (43)	2,601 (40)	**0.001**
Black	1,209 (16)	839 (13)	**< 0.001**
Hispanic or Latino	911 (12)	661 (10)	**0.004**
Comorbidities			
Essential (primary) hypertension	2,081 (27)	1,998 (31)	**< 0.001**
Type 2 Diabetes	824 (11)	747 (12)	0.083
Chronic Lower Respiratory Disease	976 (13)	931 (13)	0.653
Heart Failure	263 (3)	238 (4)	0.358
Chronic Kidney Disease	298 (4)	284 (4)	0.102
Nicotine Dependence	1,561 (20)	1,490 (23)	**< 0.001**

All data are presented as mean ± SD or n (%) unless otherwise specified.

*Statistically significant with p *≤* 0.05.

Findings with p-value < 0.05 were bolded to make it clear which findings were statitically significant.

### Surgical outcomes and complications

The most common surgical complications in both cohorts included ED visits, rehospitalization, deep infection, and arthrofibrosis requiring manipulation under anesthesia or lysis of adhesions (Table 3). The bicondylar group had a higher risk of deep infection (2-year RR 1.77 [1.57,2.00]; *p* < 0.001), rehospitalization (2-year RR 1.18 [1.10,1.25]; *p* < 0.001), arthrofibrosis (2-year RR 2.01 [1.72,2.35]; *p* < 0.001), and nonunion (2-year RR 2.25 [1.71,2.97]; *p* < 0.001) at all measured time points. The risk of SSI was higher in the bicondylar group at 180 days (RR 1.50 [0.92,2.46]; *p* = 0.010) and two years (RR 1.58 [1.01,2.47]; *p* = 0.044), while the risk of malunion was elevated only at two years (RR 1.67 [1.12,2.51]; *p* = 0.011). Notably, there was no significant difference in the rate of ED visits at any time point.

**Table 2:** Medical outcomes and Complications*

Complication	Unicondylar (*n* = 8,680)	Bicondylar (*n* = 6,954)	RR (95% CI)	*p*-value
Deep Vein Thrombosis				
30d	2.2%	2.9%	1.31 (1.08,1.60)	**0.006**
90d	3.1%	3.8%	1.21 (1.03,1.43)	**0.024**
180d	3.5%	4.3%	1.22 (1.04,1.42)	**0.015**
1y	3.7%	4.7%	1.27 (1.09,1.48)	**0.002**
Pulmonary Embolism				
30d	1.1%	1.5%	1.40 (1.06,1.85)	**0.018**
90d	1.5%	2.0%	1.38 (1.08,1.75)	**0.008**
180d	1.6%	2.2%	1.37 (1.09,1.72)	**0.007**
1y	1.7%	2.2%	1.33 (1.06,1.66)	**0.013**
Myocardial Infarction				
30d	0.4%	0.5%	1.40 (0.88,2.24)	0.158
90d	0.5%	0.6%	1.19 (0.79,1.81)	0.407
180d	0.6%	0.8%	1.32 (0.90,1.94)	0.151
1y	0.7%	0.9%	1.15 (0.81,1.63)	0.427
Stroke				
30d	1.2%	1.1%	0.88 (0.66,1.18)	0.383
90d	1.7%	1.6%	0.92 (0.72,1.18)	0.923
180d	1.9%	1.9%	0.98 (0.78,1.23)	0.875
1y	2.1%	2.1%	0.99 (0.80,1.23)	0.950
Cardiac Arrest				
30d	0.2%	0.2%	1.15 (0.53,2.52)	0.723
90d	0.2%	0.3%	1.31 (0.70,2.46)	0.392
180d	0.2%	0.3%	1.50 (0.83,2.71)	0.179
1y	0.3%	0.4%	1.29 (0.77,2.17)	0.330
Pneumonia				
30d	1.7%	2.2%	1.27 (1.02,1.59)	**0.033**
90d	2.3%	2.8%	1.22 (1.01,1.49)	**0.043**
180d	2.7%	3.1%	1.18 (0.98.1.41)	0.079
1y	3.3%	3.6%	1.09 (0.92,1.28)	0.337
Acute Kidney Injury				
30d	1.6%	2.0%	1.24 (0.98,1.56)	0.069
90d	2.1%	2.7%	1.28 (1.04,1.56)	**0.018**
180d	2.5%	3.2%	1.30 (1.08,1.57)	**0.005**
1y	3.1%	3.9%	1.25 (1.06,1.48)	**0.008**
Urinary Tract Infection				
30d	1.8%	2.0%	1.11 (0.88,1.40)	0.373
90d	2.7%	2.8%	1.03 (0.85,1.24)	0.762
180d	3.2%	3.6%	1.10 (0.93,1.30)	0.262
1y	4.0%	4.4%	1.11 (0.95,1.29)	0.176
Acute Respiratory Distress Syndrome				
30d	0.4%	0.2%	0.62 (0.34,1.14)	0.120
90d	0.4%	0.3%	0.73 (0.43,1.25)	0.245
180d	0.5%	0.4%	0.80 (0.49,1.32)	0.382
1y	0.5%	0.4%	0.77 (0.47,1.26)	0.299
Sepsis				
30d	0.7%	0.9%	1.29 (0.91,1.83)	0.152
90d	1.2%	1.6%	1.31 (1.00,1.71)	**0.049**
180d	1.5%	2.1%	1.40 (1.11,1.77)	**0.005**
1y	1.9%	2.6%	1.36 (1.11,1.68)	**0.004**
Death				
30d	0.4%	0.5%	1.32 (0.83,2.10)	0.237
90d	0.7%	0.9%	1.37 (0.97,1.95)	0.073
180d	1.0%	1.4%	1.44 (1.08,1.93)	**0.013**
1y	1.4%	2.1%	1.46 (1.15,1.86)	**0.002**

All data are presented as % unless otherwise specified

*Statistically significant with p *≤* 0.05

CI, confidence interval; RR, relative risk

Findings with p-value < 0.05 were bolded to make it clear which findings were statitically significant.

### Conversion TKA

Bivariate analysis revealed no difference in the rate of conversion to TKA at 1 year (0.4% vs. 0.4%; RR 1.11 [0.68,1.79]; *p* = 0.683); however, the risk was significantly higher in the bicondylar group at 2 years (1.4% vs. 1.0%; RR 1.39 [1.05,1.86]; *p* = 0.023), 5 years (2.3% vs., 1.6%; RR 1.43 [1.14,1.79]; *p* = 0.002), and 10 years (2.7% vs. 1.9%; RR 1.40 [1.14,1.72]; *p* = 0.001) postoperatively (Table 4). After multivariate analysis accounting for fracture type (unicondylar vs. bicondylar), age, sex, and comorbidities, bicondylar fracture remained a significant predictor of conversion to TKA at 5-year (hazard ratio (HR) 1.36 [1.08,1.71]; *p* = 0.009) and 10-year (HR 1.36 [1.10,1.69]; *p* = 0.005) follow-up (Table 5).

**Table 3:** Surgical outcomes and complications*

Complication	Unicondylar (*n* = 8,680)	Bicondylar (*n* = 6,954)	RR (95% CI)	*p*-value
Superficial Skin Infection				
30d	0.2%	0.2%	1.25 (0.60,2.62)	0.556
90d	0.4%	0.3%	1.65 (0.95,2.86)	0.075
180d	0.3%	0.5%	1.50 (0.92,2.46)	**0.010**
1y	0.4%	0.6%	1.51 (0.96,2.40)	0.076
2y	0.4%	0.6%	1.58 (1.01, 2.47)	**0.044**
Deep Infection				
30d	1.2%	2.2%	1.80 (1.41,2.30)	**< 0.001**
90d	2.2%	4.3%	1.96 (1.64,2.33)	**< 0.001**
180d	3.1%	6.0%	1.92 (1.66,2.23)	**< 0.001**
1y	4.3%	7.7%	1.79 (1.58,2.04)	**< 0.001**
2y	4.9%	8.7%	1.77 (1.57,2.00)	**< 0.001**
ED Visit				
30d	6.2%	5.9%	0.95 (0.84,1.08)	0.454
90d	10.3%	10.5%	1.02 (0.93,1.12)	0.661
180d	13.2%	13.6%	1.03 (0.95,1.11)	0.521
1y	17.0%	17.5%	1.03 (0.96,1.10)	0.491
2y	21.9%	22.3%	1.02 (0.96,1.08)	0.471
Rehospitalization				
30d	12.0%	13.8%	1.15 (1.06,1.25)	**< 0.001**
90d	13.6%	15.8%	1.16 (1.08,1.25)	**< 0.001**
180d	14.6%	17.2%	1.18 (1.09,1.27)	**< 0.001**
1y	16.0%	19.0%	1.19 (1.11,1.27)	**< 0.001**
2y	18.0%	21.1%	1.18 (1.10,1.25)	**< 0.001**
Arthrofibrosis				
30d	N/A**	N/A**	N/A**	N/A**
90d	1.2%	2.7%	2.29 (1.81,2.91)	**< 0.001**
180d	2.1%	4.3%	2.07 (1.96,2.43)	**< 0.001**
1y	2.6%	5.3%	2.06 (1.75,2.43)	**< 0.001**
2y	2.8%	5.6%	2.01 (1.72,2.35)	**< 0.001**
Malunion				
30d	N/A**	N/A**	N/A**	N/A**
90d	N/A**	N/A**	N/A**	N/A**
180d	0.3%	0.4%	1.20 (0.71,2.04)	0.491
1y	0.4%	0.6%	1.48 (0.96,2.27)	0.074
2y	0.5%	0.8%	1.67 (1.12,2.51)	**0.011**
Nonunion				
30d	N/A**	N/A**	N/A**	N/A**
90d	N/A**	N/A**	N/A**	N/A**
180d	0.5%	1.0%	1.97	**< 0.001**
1y	1.8%	0.8%	2.31 (1.71,3.11)	**< 0.001**
2y	2.0%	0.9%	2.25 (1.71,2.97)	**< 0.001**

All data are presented as % unless otherwise specified

*Statistically significant with p *≤* 0.05.

** Outcome not evaluated at given time point

CI, confidence interval; N/A, not applicable; RR, relative risk

Findings with p-value < 0.05 were bolded to make it clear which findings were statitically significant.

**Table 4:** Conversion TKA rates*

Follow-Up	Unicondylar (*n* = 8,680)	Bicondylar (*n* = 6,954)	RR (95% CI)	*p*-value
1y	0.4%	0.4%	1.11 (0.68,1.79)	0.683
2y	1.0%	1.4%	1.39 (1.05,1.86)	**0.023**
5y	1.6%	2.3%	1.43 (1.14,1.79)	**0.002**
10y	1.9%	2.7%	1.40 (1.14, 1.72)	**0.001**

All data are presented as % unless otherwise specified

*Statistically significant with p *≤* 0.05

CI, confidence interval; RR, relative risk

Findings with p-value < 0.05 were bolded to make it clear which findings were statitically significant.

**Table 5:** Multivariate Conversion TKA Analysis*

Variables	2-Years		5-Years		10-Years	
	HR (95% CI)	*p*-value	HR (95% CI)	*p*-value	HR (95% CI)	*p*-value
Bicondylar vs. Unicondylar Fracture	1.32 (0.97,1.78)	0.074	1.36 (1.08,1.71)	**0.009**	1.36 (1.10,1.69)	**0.005**
Male vs. Female Sex	1.02 (1.01,1.03)	**0.006**	0.84 (0.66, 1.06)	0.140	0.78 (0.62, 0.98)	**0.031**
Age at Surgery	1.02 (1.01,1.03)	0.230	1.02 (1.01,1.03)	**< 0.001**	1.03 (1.02,1.03)	**< 0.001**
Essential (primary) Hypertension	1.20 (0.85,1.71)	0.306	1.28 (0.98,1.67)	0.065	1.11 (0.86,1.43)	0.428
Chronic Kidney Disease	1.42 (0.80,2.52)	0.229	0.94 (0.56,1.56)	0.801	0.98 (0.60,1.59)	0.930
Type 2 Diabetes	1.08 (0.70,1.66)	0.723	1.18 (0.85,1.64)	0.318	1.05 (0.77,1.45)	0.749
Heart Failure	0.98 (0.51,1.91)	0.958	0.80 (0.44,1.43)	0.443	0.92 (0.54,1.56)	0.749
Nicotine Dependence	1.22 (0.85,1.75)	0.280	1.07 (0.79,1.46)	0.664	1.00 (0.76,1.31)	0.980

*Statistically significant with p *≤* 0.05

CI, confidence interval; HR, hazard ratio

Findings with p-value < 0.05 were bolded to make it clear which findings were statitically significant.

## Discussion

While ORIF remains the standard of care for the majority of TPFs, managing high-energy bicondylar TPFs remains a challenge as these injuries frequently lead to complications including infection, nonunion, and compartment syndrome. This large retrospective database study, utilizing the TriNetX US Collaborative Network with a cohort of 15,634 patients, found that operatively manage bicondylar TPFs were associated with higher rates of medical and surgical complications, such as DVT, deep infection, and arthrofibrosis, as well as an increased risk of conversion to TKA compared to unicondylar fractures across immediate, intermediate, and long-term postoperative periods.

Bicondylar fractures result in significant tissue damage, heightening the risk of infection. Our analysis demonstrated a significantly elevated risk of deep infection in bicondylar TPFs compared to unicondylar fractures (RR 1.77 [1.57–2.00]; *p* < 0.001). This aligns with the increased injury severity and soft tissue disruption inherent to bicondylar fractures, which frequently require dual plating and extensive surgical exposure [6]. Grisdela et al.emphasized that open, complete articular TPFs, including bicondylar fractures, have a high incidence of wound complications and frequently require soft tissue coverage [15]. Reinforcing this notion, Ruffolo et al.and Morris et al.reported a 23.6% deep infection rate in bicondylar fractures treated with dual incisions and a 14.2% deep infection rate requiring irrigation and debridement, respectively [10, 16]. Bullock et al.conducted a systematic review and meta-analysis, reporting deep infection rates of approximately 6% in unicondylar TPFs and 9% in bicondylar fractures [17]. This deep infection rate complements our rate of 8.7% over a two year period in addition to recent studies with reported deep infection rates of 7.8% to 8.8% [11, 18]. These findings emphasize the need for meticulous soft tissue management and possibly staged procedures, as advocated by Egol et al., who demonstrated reduced infection rates with temporary external fixation prior to definitive ORIF [19]. When treating bicondylar fractures, clinicians should prioritize infection prevention by developing a thoughtful and intentional approach to soft tissue management.

Bicondylar TPFs exhibited a markedly higher risk of arthrofibrosis requiring intervention (2-year RR 2.01 [1.72–2.35]; *p* < 0.001), a finding consistent from 90 days through 2 years. This contrasts with unicondylar fractures, where rates remained markedly lower, likely due to less extensive joint disruption and soft tissue trauma. Haller et al.reported a 14.5% incidence of arthrofibrosis requiring intervention in TPFs treated with ORIF; however, the study did not distinguish between unicondylar and bicondylar fractures [20]. While the exact incidence of arthrofibrosis in unicondylar versus bicondylar fractures is not distinctly reported, literature comparisons suggest that bicondylar fractures are associated with an increased likelihood of repeat ORIF and additional interventions, including greater overall complication risks [21]. Similarly, Unno et al.documented a 15.7% reoperation rate for bicondylar fractures treated with early definitive ORIF, with complications such as joint stiffness [3]. In their finite element analysis of coronal splits Samsami et al.suggest that the biomechanical instability of bicondylar fractures could contribute to postoperative stiffness by disrupting joint kinematics [7]. Ultimately, our data suggest that arthrofibrosis represents both an immediate postoperative challenge and a sustained long-term risk, contributing to ongoing joint stiffness and patient morbidity.

Rehospitalization rates were consistently elevated in the bicondylar cohort (2-year RR 1.18 [95% CI 1.10–1.25]; *p* < 0.001), reflecting a broader postoperative burden. This aligns with higher rates of deep infection, arthrofibrosis, and nonunion (2-year RR 2.25, *p* < 0.001), which collectively contribute to secondary interventions. Le Baron et al.’s comparison of assisted reduction and internal fixation (ARIF) and ORIF found no significant difference in reoperation rates; however, their analysis grouped various TPF subtypes together, which may have obscured risks specific to bicondylar fractures [2]. Our results suggest that the increased complexity of bicondylar fixation elevates the likelihood of complications necessitating hospital readmission. Ruffolo et al.’s 27.9% major complication rate in bicondylar fractures corroborates this, with infection and nonunion as key drivers [10]. Notably, ED visits showed no significant difference (2-year RR 1.02, *p* = 0.471), indicating that rehospitalizations may stem from more severe rather than minor issues. This heightened need for hospital readmissions underscores the greater resource burden and financial implications associated with bicondylar fractures, as these patients require more intensive postoperative care and management compared to their unicondylar counterparts [22].

The increased risks of DVT and PE in bicondylar TFPs (1-year RR 1.27, *p* = 0.002; RR 1.33, *p* = 0.013) compared to unicondylar fractures highlight a fracture-type-dependent susceptibility to thrombotic events, likely driven by greater immobility and surgical trauma in the bicondylar group. Milenkovic et al.analyzed 41 patients with lateral TPFs (Schatzker I-III) who underwent ORIF and identified a 2.43% incidence of non-lethal PE; however, the study did not distinguish between unicondylar and bicondylar fractures [23]. Existing literature offers some understanding of overall complication rates, including venous thromboembolism, but lacks a direct comparison of PE rates between unicondylar and bicondylar fractures. The higher prevalence of hypertension in our bicondylar group (31% vs. 27%, *p* < 0.001) might suggest a cardiovascular predisposition, yet this did not translate to significant events. These findings underscore the importance of DVT prophylaxis as supported by recent literature on post-discharge strategies to mitigate thromboembolism risk in patients with tibial plateau fractures [23–25] Larger studies or extended follow-up could further elucidate these patterns, but our data emphasize the need for vigilant thromboprophylaxis in bicondylar fracture patients.

The increased incidence of conversion TKA amongst bicondylar fractures at 2 years (RR 1.39, *p* = 0.023), 5 years (RR 1.43, *p* = 0.002), and 10 years (RR 1.40, *p* = 0.001), reflects a trajectory of progressive joint degeneration. Multivariate analysis further reinforced these findings, with bicondylar fracture persisting as an independent predictor at 5 and 10 years. Wasserstein et al.reported a 7.3% 10-year conversion TKA rate across all TPFs, though without subtype differentiation, while Kraml et al.reported a rate of 12.2% [12, 13]. The lack of significant differences at early time points reflects the idea that initial joint stability achieved via ORIF may deteriorate due to cartilage damage and malalignment, more pronounced in bicondylar fractures. Gupta et al.found that severe joint depression in patients over 60 years old significantly predicts TKA, alluding to a synergy between fracture severity and age-related cartilage vulnerability [4]. Our findings advocate for long-term monitoring of bicondylar patients, with early intervention potentially delaying conversion TKA. However, the relatively lower rates observed in our study compared to prior reports may reflect the TriNetX database’s inclusion of a more diverse patient cohort and range of practice environments than those typically represented in the literature.

This study has limitations that warrant consideration. The reliance on CPT and ICD-10 coding within the TriNetX database precluded detailed stratification of TPFs by specific morphological characteristics. The retrospective nature of this analysis, while leveraging a large cohort of patients, inherently limits the ability to establish causality and restricts the findings to associations rather than definitive evidence for clinical decision-making. Additionally, the accuracy and completeness of the data depend on proper coding by healthcare providers, and without direct access to individual patient records, potential misclassifications or omissions could affect the results. Although multivariate analysis adjusted for age, sex, and select comorbidities, unmeasured confounders may still influence the outcomes. Despite these constraints, this study provides valuable insights into the comparative postoperative trajectories of unicondylar and bicondylar TPFs.

## Conclusion

Patients undergoing operative fixation of bicondylar tibial plateau fractures experience higher rates of medical and surgical complications, as well as an increased incidence of conversion to TKA, compared to those with unicondylar fractures. While ORIF remains the cornerstone of treatment for TPFs, these elevated risks accentuate the challenges of managing the greater complexity and injury severity inherent to bicondylar fractures. These findings emphasize the need for precise surgical techniques, individualized patient management, and robust postoperative monitoring, while demonstrating the importance of targeted thromboprophylaxis and early intervention strategies to reduce complications and optimize long-term joint outcomes.

## Supplementary Information

Below is the link to the electronic supplementary material.

Supplementary Material 1

## Data Availability

No datasets were generated or analysed during the current study.
