# Supplementary material for: Complications and TKA conversion rates in unicondylar vs. bicondylar tibial plateau fractures: a retrospective cohort analysis
Source: Arch Orthop Trauma Surg. 2025 Oct 29;145(1):491. doi: 10.1007/s00402-025-06107-0 (PMC12572020; doi:10.1007/s00402-025-06107-0)
Supplement: Supplementary file 1 — Supplementary Material 1 [file 402_2025_6107_MOESM1_ESM.docx]

| **Supplementary Table 1.** ICD and CPT codes used to query TriNetX platform and generate patient cohorts |
| --- |
| - Unicondylar tibial plateau fracture   - Open treatment of tibial fracture, proximal (plateau); unicondylar, includes internal fixation, when performed (CPT 27535) - Bicondylar tibial plateau fracture   - Open treatment of tibial fracture, proximal (plateau); bicondylar with or without internal fixation (CPT 27536) - Total knee arthroplasty   - Arthroplasty, knee, condyle and plateau; medial AND lateral compartments with or without patella resurfacing (total knee arthroplasty) (CPT 27447) - Deep vein thrombosis   - Acute embolism and thrombosis of deep veins of lower extremity (UMLS:ICD10CM:I82.4) - Urinary tract infection   - Acute cystitis  (UMLS:ICD10CM:N30.0)   - Urinary tract infection, site not specified  (UMLS:ICD10CM:N39.0)   - Infection and inflammatory reaction due to urinary catheter  (UMLS:ICD10CM:T83.51) - Pulmonary embolism   - Pulmonary embolism (UMLS:ICD10CM:I26) - Myocardial infarction   - Acute myocardial infarction (UMLS:ICD10CM:I21) - Stroke   - Cerebral infarction (UMLS:ICD10CM:I63)   - Nontraumatic intracerebral hemorrhage (UMLS:ICD10CM:I61)   - Nontraumatic subarachnoid hemorrhage (UMLS:ICD10CM:I60) - Cardiac arrest   - Cardiac arrest (UMLS:ICD10CM:I46) - Pneumonia   - Viral pneumonia, not elsewhere classified (UMLS:ICD10CM:J12)   - Pneumonia due to streptococcus pneumoniae (UMLS:ICD10CM:J13)   - Pneumonia due to Hemophilus influenzae (UMLS:ICD10CM:J14)   - Bacterial pneumonia, not elsewhere classified (UMLS:ICD10CM:J15)   - Pneumonia due to other infectious organisms, not elsewhere classified (UMLS:ICD10CM:J16)   - Pneumonia in diseases classified elsewhere (UMLS:ICD10CM:J17)   - Pneumonia, unspecified organism (UMLS:ICD10CM:J18) - Acute kidney injury   - Acute kidney failure (UMLS:ICD10CM:N17) - Acute respiratory distress syndrome   - Acute respiratory distress syndrome (UMLS:ICD10CM:J80) - Sepsis   - Other sepsis (UMLS:ICD10CM:A41)   - Severe sepsis (UMLS:ICD10CM:R65.2) - Superficial skin infection   - Infection following a procedure, superficial incisional surgical site (UMLS:ICD10CM:T81.41) - Deep injection/prosthetic joint infection   - Infection following a procedure, deep incisional surgical site (UMLS:ICD10CM:T81.42)   - Infection and inflammatory reaction due to internal joint prothesis (UMLS:ICD10CM:T84.5)   - Infection and inflammatory reaction to internal fixation device (UMLS:ICD10CM:T84.6)   - Infection and inflammatory reaction due to other internal orthopedic prosthetic devices, implants and grafts (UMLS:ICD10CM:T84.7)   - Arthroscopy, knee, surgical; for infection, lavage and drainage (CPT 29871)   - Arthrotomy, knee, with exploration, drainage, or removal of foreign body (eg, infection) (CPT 27310)   - Incision and drainage, deep abscess, bursa, or hematoma, thigh or knee region (CPT 27301) - ED visits   - Emergency department visit for the evaluation and management of a patient hat may not require the presence of a physician or other qualified health care professional (CPT 99281)   - Emergency department visit for the evaluation and management of a patient, which requires a medically appropriate history and/or examination and straightforward medical decision making (CPT 99282)   - Emergency department visit for the evaluation and management of a patient, which requires a medically appropriate history and/or examination and moderate level of medical decision making (CPT 99284)   - Emergency department visit for the evaluation and management of a patient, which requires a medically appropriate history and/or examination and high level of medical decision making (CPT 99285)   - Physician or other qualified health care professional direction of emergency medical systems (EMS) emergency care, advanced life support (CPT 99288) - Rehospitalization   - Initial hospital or observation care, per day, for the evaluation and management of a patient, which requires a medically appropriate history and/or examination and high level of medical decision making. When using total time on the date of the encounter for code selection, 75 minutes must be met or exceeded (CPT 99223)   - Initial hospital or observation care, per day, for the evaluation and management of a patient, which requires a medically appropriate history and/or examination and moderate level of medical decision making. When using total time on the date of the encounter for code selection, 55 minutes must be met or exceeded (CPT 99222)   - Initial hospital or observation care, per day, for the evaluation and management of a patient, which requires a medically appropriate history and/or examination and straightforward or low level of medical decision making. When using total time on the date of the encounter for code selection, 40 minutes must be met or exceeded (CPT 99221) - Nonunion   - Displaced bicondylar fracture of right tibia, subsequent encounter for closed fracture with nonunion (UMLS:ICD10CM:S82.141K)   - Displaced bicondylar fracture of right tibia, subsequent encounter for open fracture type I or II with nonunion (UMLS:ICD10CM:S82.141M)   - Displaced bicondylar fracture of right tibia, subsequent encounter for open fracture type IIIA or IIIB, or IIIC with nonunion (UMLS:ICD10CM:S82.141N)   - Displaced bicondylar fracture of left tibia, subsequent encounter for closed fracture with nonunion (UMLS:ICD10CM:S82.142K)   - Displaced bicondylar fracture of left tibia, subsequent encounter for open fracture type I or II with nonunion (UMLS:ICD10CM:S82.142M)   - Displaced bicondylar fracture of left tibia, subsequent encounter for open fracture type IIIA or IIIB, or IIIC with nonunion (UMLS:ICD10CM:S82.142N)   - Displaced bicondylar fracture of unspecified tibia, subsequent encounter for closed fracture with nonunion (UMLS:ICD10CM:S82.143K)   - Displaced bicondylar fracture of unspecified tibia, subsequent encounter for open fracture type I or II with nonunion (UMLS:ICD10CM:S82.143M)   - Displaced bicondylar fracture of unspecified tibia, subsequent encounter for open fracture type IIIA or IIIB, or IIIC with nonunion (UMLS:ICD10CM:143N)   - Nondisplaced bicondylar fracture of right tibia, subsequent encounter for closed fracture with nonunion (UMLS:ICD10CM:S82.144K)   - Nondisplaced bicondylar fracture of right tibia, subsequent encounter for open fracture type I or II with nonunion (UMLS:ICD10CM:S82.144M)   - Nondisplaced bicondylar fracture of left tibia, subsequent encounter for closed fracture with nonunion (UMLS:ICD10CM:S82.145K)   - Nondisplaced bicondylar fracture of left tibia, subsequent encounter for open fracture type I or II with nonunion (UMLS:ICD10CM:S82.145M)   - Nondisplaced bicondylar fracture of unspecified tibia, subsequent encounter for closed fracture with nonunion (UMLS:ICD10CM:S82.146K)   - Displaced fracture of lateral condyle of right tibia, subsequent encounter for closed fracture with nonunion (UMLS:ICD10CM:S82.121K)   - Displaced fracture of lateral condyle of right tibia, subsequent encounter for open fracture type I or II with nonunion (UMLS:ICD10CM:S82.121M)   - Displaced fracture of lateral condyle of right tibia, subsequent encounter for open fracture type IIIA or IIIB, or IIIC with nonunion (UMLS:ICD10CM:S82.121N)   - Displaced fracture of lateral condyle of left tibia, subsequent encounter for closed fracture with nonunion (UMLS:ICD10CM:S82.122K)   - Displaced fracture of lateral condyle of left tibia, subsequent encounter for open fracture type I or II with nonunion (UMLS:ICD10CM:S82.122M)   - Displaced fracture of lateral condyle of left tibia, subsequent encounter for open fracture type IIIA or IIIB, or IIIC with nonunion (UMLS:ICD10CM:S82.122N)   - Displaced fracture of lateral condyle of unspecified tibia, subsequent encounter for closed fracture with nonunion (UMLS:ICD10CM:S82.123K)   - Nondisplaced fracture of lateral condyle of right tibia, subsequent encounter for closed fracture with nonunion (UMLS:ICD10CM:S82.124K)   - Nondisplaced fracture of lateral condyle of right tibia, subsequent encounter for open fracture type I or II with nonunion (UMLS:ICD10CM:S82.124M)   - Nondisplaced fracture of lateral condyle of left tibia, subsequent encounter for closed fracture with nonunion (UMLS:ICD10CM:S82.125K)   - Nondisplaced fracture of lateral condyle of unspecified tibia, subsequent encounter for closed fracture with nonunion (UMLS:ICD10CM:S82.126K)   - Displaced fracture of medial condyle of right tibia, subsequent encounter for closed fracture with nonunion (UMLS:ICD10CM:S82.131K)   - Displaced fracture of medial condyle of right tibia, subsequent encounter for open fracture type I or II with nonunion (UMLS:ICD10CM:S82.131M)   - Displaced fracture of medial condyle of right tibia, subsequent encounter for open fracture type IIIA or IIIB, or IIIC with nonunion (UMLS:ICD10CM:S82.131N)   - Displaced fracture of medial condyle of left tibia, subsequent encounter for closed fracture with nonunion (UMLS:ICD10CM:S82.132K)   - Displaced fracture of medial condyle of left tibia, subsequent encounter for open fracture type I or II with nonunion (UMLS:ICD10CM:S82.132M)   - Displaced fracture of medial condyle of unspecified tibia, subsequent encounter for closed fracture with nonunion (UMLS:ICD10CM:S82.133K)   - Displaced fracture of medial condyle of unspecified tibia, subsequent encounter for open fracture type I or II with nonunion (UMLS:ICD10CM:S82.133N)   - Displaced fracture of medial condyle of unspecified tibia, subsequent encounter for open fracture type IIIA or IIIB, or IIIC with nonunion (UMLS:ICD10CM:S82   - Nondisplaced fracture of medial condyle of left tibia, subsequent encounter for closed fracture with nonunion (UMLS:ICD10CM:S82.135K) - Malunion   - Displaced fracture of lateral condyle of right tibia, subsequent encounter for closed fracture with malunion (UMLS:ICD10CM:S82.121P)   - Displaced fracture of lateral condyle of right tibia, subsequent encounter for open fracture type I or II with malunion (UMLS:ICD10CM:S82.121Q)   - Displaced fracture of lateral condyle of right tibia, subsequent encounter for open fracture type IIIA, IIIB, or IIIC with malunion (UMLS:ICD10CM:S82.121R)   - Displaced fracture of lateral condyle of left tibia, subsequent encounter for closed fracture with malunion (UMLS:ICD10CM:S82.122P)   - Displaced fracture of lateral condyle of left tibia, subsequent encounter for open fracture type I or II with malunion (UMLS:ICD10CM:S82.122Q)   - Displaced fracture of lateral condyle of unspecified tibia, subsequent encounter for closed fracture with malunion (UMLS:ICD10CM:S82.123P)   - Displaced fracture of lateral condyle of unspecified tibia, subsequent encounter for open fracture type I or II with malunion (UMLS:ICD10CM:S82.123Q)   - Nondisplaced fracture of lateral condyle of right tibia, subsequent encounter for closed fracture with malunion (UMLS:ICD10CM:S82.124P)   - Nondisplaced fracture of lateral condyle of left tibia, subsequent encounter for closed fracture with malunion (UMLS:ICD10CM:S82.125P)   - Nondisplaced fracture of lateral condyle of unspecified tibia, subsequent encounter for closed fracture with malunion (UMLS:ICD10CM:S82.126P)   - Displaced fracture of medial condyle of right tibia, subsequent encounter for closed fracture with malunion (UMLS:ICD10CM:S82.131P)   - Displaced fracture of medial condyle of right tibia, subsequent encounter for open fracture type I or II with malunion (UMLS:ICD10CM:S82.131Q)   - Displaced fracture of medial condyle of right tibia, subsequent encounter for open fracture type IIIA, IIIB, or IIIC with malunion (UMLS:ICD10CM:S82.131R)   - Displaced fracture of medial condyle of left tibia, subsequent encounter for closed fracture with malunion (UMLS:ICD10CM:S82.132P)   - Displaced fracture of medial condyle of left tibia, subsequent encounter for open fracture type I or II with malunion (UMLS:ICD10CM:S82.132Q)   - Displaced fracture of medial condyle of left tibia, subsequent encounter for open fracture type IIIA, IIIB, or IIIC with malunion (UMLS:ICD10CM:S82.132R)   - Displaced fracture of medial condyle of unspecified tibia, subsequent encounter for closed fracture with malunion (UMLS:ICD10CM:S82.133P)   - Nondisplaced fracture of medial condyle of right tibia, subsequent encounter for closed fracture with malunion (UMLS:ICD10CM:S82.134P)   - Nondisplaced fracture of medial condyle of left tibia, subsequent encounter for closed fracture with malunion (UMLS:ICD10CM:S82.135P)   - Nondisplaced fracture of medial condyle of unspecified tibia, subsequent encounter for closed fracture with malunion (UMLS:ICD10CM:S82.136P)   - Displaced bicondylar of right tibia, subsequent encounter for closed fracture with malunion (UMLS:ICD10CM:S82.141P)   - Displaced bicondylar of right tibia, subsequent encounter for open fracture type I or II with malunion (UMLS:ICD10CM:S82.141Q)   - Displaced bicondylar of right tibia, subsequent encounter for open fracture type IIIA, IIIB, or IIIC with malunion (UMLS:ICD10CM:S82.141R)   - Displaced bicondylar of left tibia, subsequent encounter for closed fracture with malunion (UMLS:ICD10CM:S82124P)   - Displaced bicondylar of left tibia, subsequent encounter for open fracture type I or II with malunion (UMLS:ICD10CM:S82.142Q)   - Displaced bicondylar of left tibia, subsequent encounter for open fracture type IIIA, IIIB, or IIIC with malunion (UMLS:ICD10CM:S82.142R)   - Displaced bicondylar of unspecified tibia, subsequent encounter for closed fracture with malunion (UMLS:ICD10CM:S82.143P)   - Displaced bicondylar of unspecified tibia, subsequent encounter for open fracture type I or II with malunion (UMLS:ICD10CM:S82.143Q)   - Displaced bicondylar of unspecified tibia, subsequent encounter for open fracture type IIIA, IIIB, or IIIC with malunion (UMLS:ICD10CM:S82.143R)   - Nondisplaced bicondylar of right tibia, subsequent encounter for closed fracture with malunion (UMLS:ICD10CM:S82.144P)   - Nondisplaced bicondylar of left tibia, subsequent encounter for closed fracture with malunion (UMLS:ICD10CM:S82.145P)   - Nondisplaced bicondylar of unspecified tibia, subsequent encounter for closed fracture with malunion (UMLS:ICD10CM:S82.146P) - Arthrofibrosis   - Arthroscopy, knee, surgical; with lysis of adhesions, with or without manipulation (CPT 29884)   - Manipulation of knee joint under general anesthesia (includes application of traction or other fixation devices) (CPT 27570) |
| Abbreviations: CPT, Current Procedural Terminology; ICD10, International Classification of Disease, 10th Revision |
